# Nanoparticles in intraocular drug delivery

**DOI:** 10.1515/medgen-2024-2058

**Published:** 2025-02-12

**Authors:** Stefanie E. Klaus, Miriam Breunig, Achim Göpferich

**Affiliations:** University of Regensburg Department of Pharmaceutical Technology Universitätsstr. 31 93053 Regensburg Germany; University of Regensburg Department of Pharmaceutical Technology Universitätsstr. 31 93053 Regensburg Germany; University of Regensburg Department of Pharmaceutical Technology Universitätsstr. 31 93053 Regensburg Germany

**Keywords:** Ocular drug delivery, nanotherapy, ocular barriers, posterior segment, diabetic retinopathy, EPR effect

## Abstract

Visual impairment is a severe global health problem. Underlying ocular diseases can affect both the anterior and posterior eye. Unfortunately, the efficient delivery of drug molecules to the posterior eye, especially the retina, is still a major challenge. This review provides an overview of the difficulties and limitations of current delivery options for the treatment of eye diseases in the light of drug nanotherapy. Furthermore, ocular changes that are relevant for nanotherapeutic approaches are illustrated using the example of diabetic retinopathy (DR). The therapeutic focus is on promising approaches of using nanoparticles (NP) for the therapy of the posterior segment of the human eye. The overall aim of this review is to scrutinize how NP could fill gaps in the field of ocular drug therapy.

## Introduction

**Figure 1:**
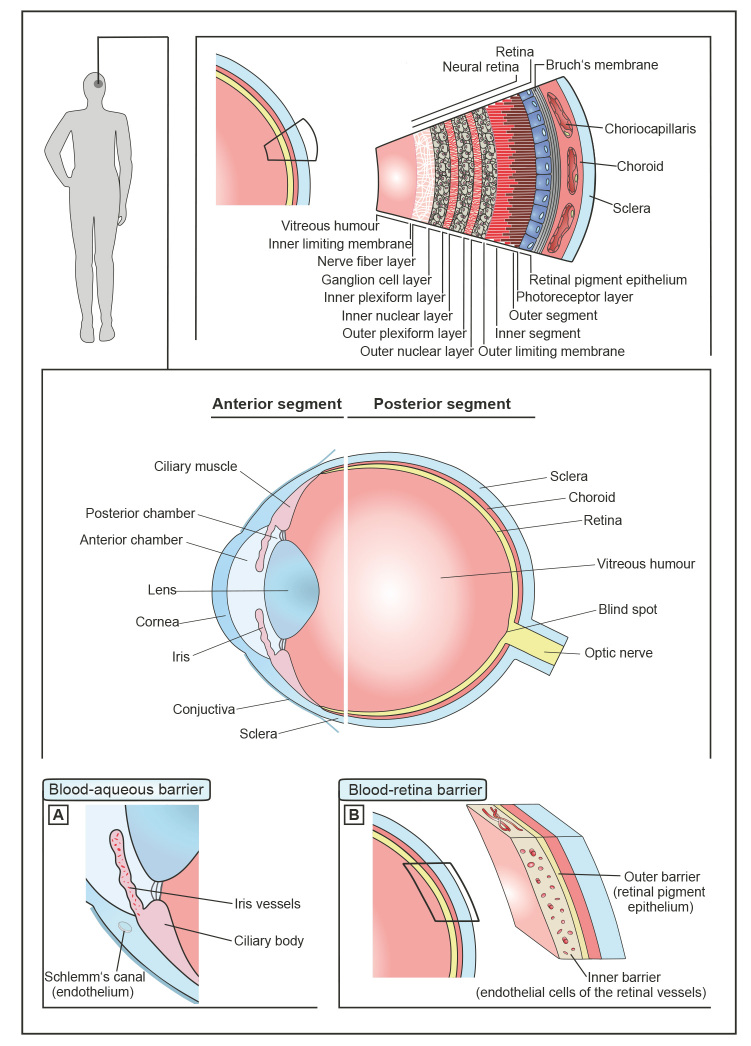
The human eye is divided into the anterior and posterior segment. The ciliary muscle, the posterior and anterior chamber, the lens, cornea and iris as well as the conjunctiva form the anterior part of the eye. (A) The blood-aqueous barrier is located here and controls the exchange between blood and intraocular fluids. This barrier is formed by tight junctions of the inner, non-pigmented epithelium of the ciliary body. The posterior segment consists of three outer layers – the sclera, the choroid and the retina – which surround the vitreous humour; the Bruch’s membrane separates the RPE from the choroid, which supplies the retinal cells with oxygen and nutrients. The retina itself consists of ten cell layers: Retinal pigment epithelium (RPE), photoreceptor layer (comprise an outer and inner segment), outer limiting membrane (OLM), outer nuclear layer (ONL), outer plexiform layer (OPL), inner nuclear layer (INL), ganglion cell layer (GCL), nerve fiber layer (NFL) and inner limiting membrane (ILM). In summary, the retina can be divided into two parts: the RPE forms the outer layer while the other nine layers facing the vitreous humour form the inner layer. This inner layer is also known as neural retina. (B) The blood-retina barrier (BRB) of the posterior segment is a selectively permeable barrier between the retina and its supplying vessels. Endothelial cells of the retinal blood vessels that are connected by tight junctions form the inner BRB; the outer BRB consists of tight junctions of the RPE. The BRB regulates the microenvironment of the retina and thus plays a decisive role in the health of the eye and the visual process; these blood-ocular-barriers prevent uncontrolled diffusion of molecules to protect the retina and thus maintain vision, but also limit free passage of ophthalmic and systemically administered drugs to the retina and thus make the administration of drug molecules to retinal cells a challenge.

Eye diseases can impair vision to the point of blindness. According to the WHO, 30 million people worldwide are currently blind, and 246 million people are visually impaired. Thereby, diseases of the eye range from localized injuries to significant and serious changes in the very complex structure of the sensory organ. Most cases of blindness are due to diseases of the anterior segment of the human eye [1]. This also includes cataract, the main cause of blindness worldwide [2]. Diseases affecting the posterior segment include DR [3] and age-related macular degeneration (AMD) [4]. For example, of the estimated 1.3 billion diabetic by 2050 [5], 27 % will develop DR which is currently already causing 3.7 million cases of visual impairment and more than 800.000 cases of blindness [6]. DR, which is also associated with microvascular complications of intraretinal blood vessels, is in addition one of several retinal disease for which the retinal pigment epithelium (RPE), a cell monolayer underlying the neuroretina, is massively involved in disease pathophysiology [7]. Apart from DR, the cell monolayer plays a pivotal role for AMD [8], retinopathy of prematurity [9], vitelli-form maculopathies [10], retinitis pigmentosa [11] and Stargardt disease [12]. Enormous efforts to deliver therapeutic compounds to the posterior segment of the eye following systemic administration via oral or parenteral routes of application have not led to the desired success. The delivery of drugs to the retina remains a major challenge. This is mainly due to the complex anatomy and physiology of the human eye (figure 1). Ocular tissue layers such as Bruch’s membrane, as well as the inner and outer blood-retina-barrier (BRB) formed by the endothelial cells of retinal capillaries and the RPE monolayer, respectively, represent a major hurdle for systemically administered drug molecules on their way to the retina. However, disease related changes in the structure and integrity of these barriers are possible. The properties of Bruch’s membrane, for example, are changed by AMD pathogenesis [13]. The integrity of the BRB is challenged by hyperglycemia-induced oxidative stress, which contributes to the development of DR via a number of biochemical mechanisms [14]. Ultimately, these changes may have an impact on the administration of therapeutic molecules, especially to the posterior segment of the eye [15–23].

## Nanoparticles as a tool to overcome limits of retinal drug therapy

**Figure 2:**
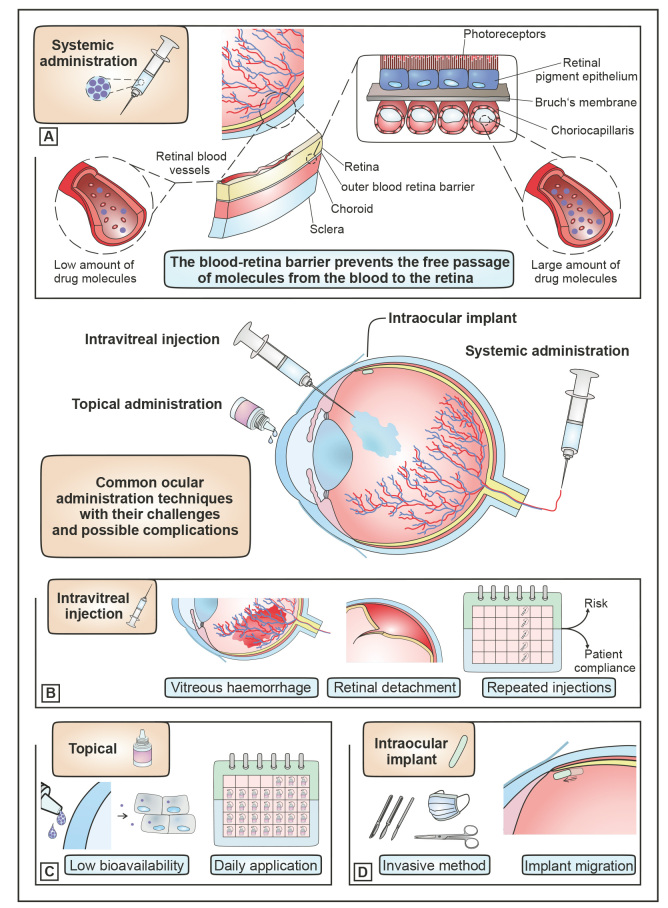
Common application routes for ocular therapy are intravitreal injection, topical and systemic administration or injection/implantation of intraocular implants. (A) For treatment of the posterior eye, drug molecules can be administered intravenously. To reach the RPE, drug molecules must first leave the blood vessels of the choroid (choriocapillaris) and then cross the Bruch’s membrane. However, the BRB between the retina and the vessels supplying the retina prevents free passage of molecules. Thus, this barrier limits the effectiveness of intravenously administered drugs and only a small fraction of molecules reaches retinal blood vessels. (B) Intravitreal injections administer the medication directly into the vitreous humour. It may cause a number of serious complications such as vitreous haemorrhage and retinal detachment. Repeated administration is required to maintain therapeutic drug concentrations, which again increases the risk of side effects and reduces patient compliance. (C) The bioavailability of topically applied drugs is reduced by various barriers such as lacrimal gland, tear dilution and conjunctival absorption. Multiple daily applications are required to maintain therapeutic drug levels at the site of action. (D) Intraocular implantation or injection of drug devices into the posterior segment of the eye is invasive. Serious phenomena such as implant migration are possible and require further and sometimes surgical interventions.

The various common application methods for ocular drug therapy with their respective challenges and limitations are shown in figure 2. Possible application routes for ocular therapy comprise topical and systemic administration, intravitreal injections, and intraocular implants. The most common and preferred method is topical administration in the form of eye drops [24]. The challenge for an efficient treatment of this simple, safe and well-tolerated way of application is the low bioavailability of less than 5 % of the administered drug [25]. Ocular barriers can reduce the precorneal residence time and cause low drug concentrations at the pharmacological site of action. These include static barriers such as the cornea as well as dynamic barriers like the clearance of the lacrimal fluid. In order to achieve better efficacy with drug molecules, they can be administered via intraocular injection or intraocular implants [26]. To overcome the limits of systemic and classical drug therapy via the cornea, the local administration of drugs to the eye via injections has been in the focus of ocular drug therapy in recent years. However, the therapeutic successes of the essentially invasive ways of drug administration using solutions of low molecular weight drugs have been moderate. Dexamethasone is a prominent example from the group of low molecular weight drugs, which are characterized by their small molecular weight (<500Da), their usual production via chemical synthesis as well as their physical and chemical properties [27,28]. A fundamental problem is in many cases the failure to maintain therapeutically relevant drug levels due to the fast clearance of substances from the eye. To make up for this would require a high injection frequency that suffers from low patient compliance. As a countermeasure, drug delivery systems for the continuous and controlled release of drugs in the eye are needed. NP are just one of them. In addition, there is a need for preferably less invasive treatment of chronic disease including DR. For this reason, the ultimate main goal is to overcome the protective barriers of the eye and to achieve high therapeutic efficacy without permanently damaging the tissue [17–19,29–31].

**Figure 3:**
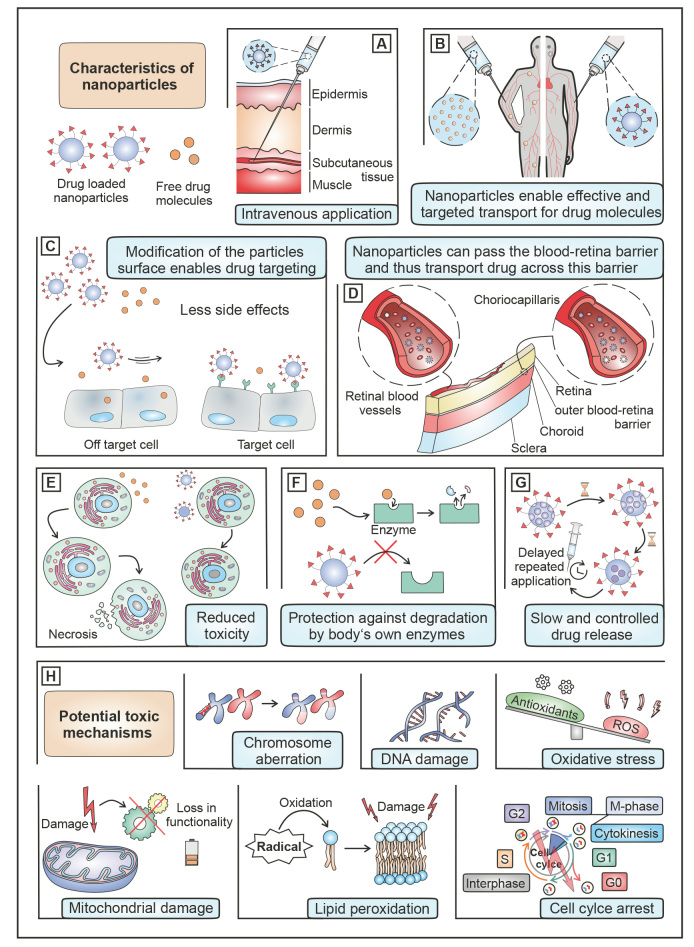
Administration of drugs that are loaded into NPs have several advantages in comparison to drugs that are applied in their free form. (A and B) After intravenous application, NPs are able to reach their target tissue while free drug substances are distributed in the entire body according to their physicochemical properties. (C) NP surface modifications enable the accumulation into target cells, while side effects are reduced. (D) In contrast to free drug molecules, NPs can pass the blood-retina barrier, which makes drug transport to the posterior eye segment possible. (E and F) The inclusion of drug molecules into NPs may reduce toxicity to cells while at the same time these molecules are protected against degradation by enzymes. (G) A slow and controlled release of entrapped molecules ensures a constant drug blood level and thus extends the time interval to the next application. (H) NP may cause toxic reactions such as chromosome aberration, DNA damage, oxidative stress, mitochondrial damage, lipid peroxidation or cell cycle arrest.

NP offer enormous possibilities to overcome some of the limitations of conventional ocular drug therapy [15,32–34]. As outlined in figure 3. Nanoparticles can increase the solubility of hydrophobic drugs [15], reduce drug toxicity [29] and protect them against degradation [24]. Loaded with drug molecules NP have the ability for sustained drug release [19], which can reduce the dosing frequency [29]. Moreover, the modification of the NP surface allows for targeting specific cell types [35,36] whereby side effects are reduced [32]. Furthermore, the penetration of drug molecules across ocular barriers is improved and overcoming the BRB leads in a better bioavailability of the drugs [15,16]. It has been shown, for example, that NPs can overcome the ILM following intraocular injection [37,38]. To efficiently target the RPE, lipoprotein mimetic lipid NP were developed that overcome the BRB. They were designed to mimic 30–80nm diameter very low-density lipoproteins NP (VLDL) which supply RPE cells with lipids and are able to cross Bruch’s membrane on their way from the choroid to the RPE. The authors could show that the NP followed the path of the VLDL particles and accumulated in the RPE where they released the incorporated drug [38]. Thereby, the cyclic RGD (arginine-glycine-aspartic acid) peptide that was tethered to the phospholipids was presented in the NP corona and allowed for hetero-multivalent target cell recognition. By modifying the NP surface with the RGD peptide as targeting ligand, the RGD-conjugated nanocarriers bind to α_v_β_3_ integrin via hydrogen bonds and metal ion coordination [39] and thus trigger internalization by RPE cells. Ultimately, the therapeutic efficacy can be improved by functionalizing NP with the peptide, whereby the cyclic RGD shows better activity compared to the linear structures, as they can resist proteolysis with their cyclic structure and have a higher affinity for integrin receptors [39,40].

In a mouse model of retinopathy of prematurity, these biomimetic particles were able to transport high amounts of cyclosporin A into the retina. One hour after intravenous (IV) administration, 2 % of the total dose of administered drug could be detected in the retina and could stop disease development in contrast to control animals receiving an IV injection of an aqueous cyclosporin A solution of the same dose. Even 5 days after their application, significant amounts of NP could still be detected inside RPE cells, indicating the formation of a NP depot in the eye. In addition, the use of these drug-loaded NPs did not cause any morphological changes in the retinae of healthy or diseased mice, which reflects their high compatibility with the tissue. Consequently, the retinal bioavailability of the therapeutic molecules is improved [38]. The example shows unequivocally that NPs can overcome the limitations of conventional drug therapy of retinal cells with low molecular weight drugs. The example also shows that the spatial and temporal distribution of NPs in the eye can be controlled by a carefully chosen particle composition and structure. It is decisive for the interactions of particles with cells and tissues. Thereby, size, shape, charge and surface properties are physicochemical parameters of paramount significance [9,19]. Consequently, nanotechnologies opens up a new perspective for the diagnosis and treatment of various (also chronic) disease [33].

## The relevance of disease pathophysiology for drug nanotherapy

DR and AMD are two examples, that drug therapy with NP could take advantage of the pathophysiological changes that come along with the disease. AMD, a degenerative retinal disease affecting the central macular area, also involves the complex of photoreceptors, RPE and Bruch’s membrane and choroid. AMD is caused by malfunction and degeneration of the RPE. A lack of cellular control of oxidative stress, altered proteostasis, disruption of lipid homeostasis and mitochondrial dysfunction contribute to the failure of the RPE. The formation of drusen is facilitated by the accumulation of abnormally misfolded proteins and abnormal lipids and as centers of inflammation. Finally, drusen can trigger chronic inflammation in the subretinal space. Inflammatory reactions occur in the context of chronic inflammation with strong involvement of the complement system and aging of the microvasculature of the choroid [42]. The thickening and reduced permeability of Bruch’s membrane causes an obstruction of nutrient transport to the retina and the excretion of waste via the choroid results in a thinning of choroidal vessels. Neovascularization, spurred by increased vascular endothelial growth factor (VEGF) levels, causes the growth of blood vessels from the choroid through Bruch‘s membrane into the retina. Leaky vessels neovascularization and subretinal and intraretinal fluids are present in wet AMD [43].

The main characteristic of diabetes mellitus (DM) is chronic hyperglycemia damaging various tissues and organs in the long run. DR, a microvascular disease, is characterized by various inflammation-related characteristics such as increased vascular flow and vascular leakage due to vascular lesions, cellular inflammation, tissue edema, expression of adhesion molecules and cytokines, reactive glia, apoptosis of inner retinal cells and neovascularization [14,44]. Increased glucose via the polyol pathway, accumulation of advanced glycation endproduct (AGE), inflammation and activation of proteinkinase C (hexosamine pathway) are biochemical metabolic pathways associated with the typical hyperglycemia-induced vascular damage. Mitochondrial dysfunction causes the production of reactive oxygen species (ROS), which increase the oxidative stress in the retina [14,45] leading to thickened basement membranes and apoptosis of pericytes. The hyperfusion caused by the loss of pericytes leads to neovascularization [14]. The newly formed blood vessels are sensitive, permeable and fragile and thus favor vitreous bleeding [46]. This type of DR pathophysiology is typical of proliferative DR (PDR) while the nonproliferative (NPDR) form is not accompanied by new vessel formation [47].

**Figure 4:**
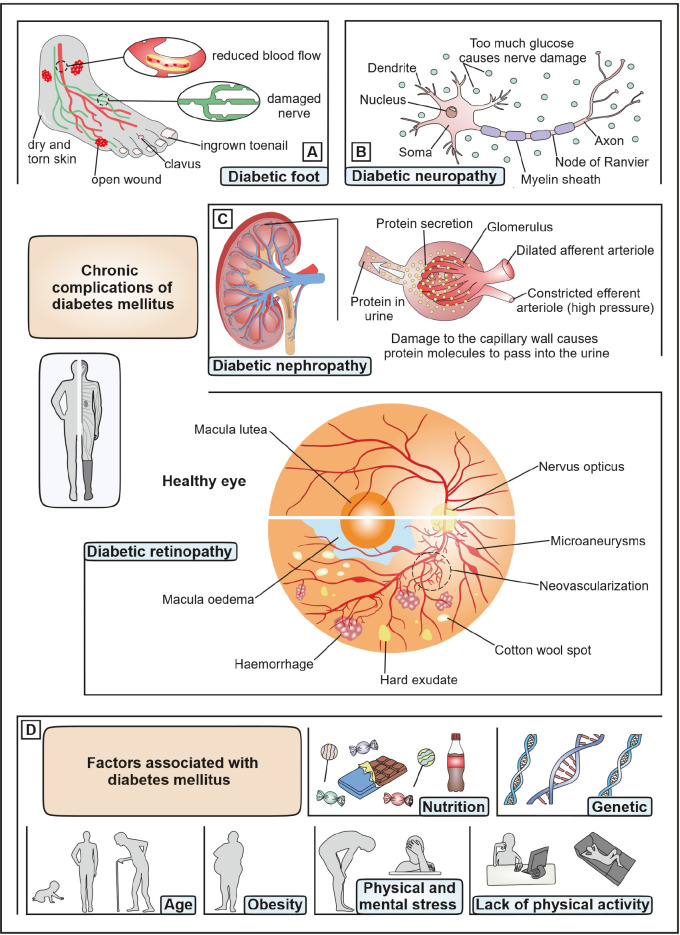
Chronic complications of diabetes mellitus. Compared to the healthy retina, retinal tissue that is affected by diabetic retinopathy shows structural changes such as macula oedema, haemorrhage, hard exudate, cotton wool spot, neovascularization, and microaneurysms. Other late manifestations of diabetes mellitus are (A) diabetic foot, (B) diabetic neuropathy and (C) diabetic nephropathy. (D) Factors associated with diseases pathogenesis are nutrition, genetics, age, obesity, physical and mental Stress and lack of physical activity.

In both cases, wet AMD and PDR, the angiogenic factors Ang1 and Ang2 together with VEGF cause an increase of vascular permeability [14] which can to some extent be reduced by the use of anti-inflammatory drugs [45,48]. However, the chronic complications of DM (figure 4) and AMD are often irreversible and place a burden on the patient’s health and overall life. These include increased vascular permeability and an accumulation of fluid in and under the retina. In addition, edema and necrosis can cause neurodegeneration that is discussed to be present even before vascular changes are observed. In addition, damage to the BRB results in ischemic changes in the surrounding retina. By activating the body’s own compensatory mechanisms, the formation and proliferation of new vessels is promoted. This makes therapeutic intervention necessary in order to control the progression of the disease [19,33,36,49]. Besides laser treatment, intravitreal injections of corticosteroid (implants) and anti-vascular endothelial growth factor (VEGF) substances such as antibodies that stop excessive blood vessel proliferation are frequent treatment modalities. However, due to the chronic nature of DR, frequent intravitreal injections are required, which may lead to serious side effects (figure 2). Especially low molecular weight drugs suffer from rapid clearance. Intraretinal implants can reduce the application frequency, but are also subject to multiple complications [19,33]. NP could help to provide an additional treatment option which would be complementary to existing treatment strategies.

Like in tumor tissue, the permeability of the excessively sprouting blood vessels in proliferative DR [50] and wet AMD [51] is increased. Changes in the cell-cell-contacts between endothelial cells make them less tight and allow macromolecules and nanoparticles to leave the blood stream and diffuse into the surrounding tissue. In tumors the drainage of lymph fluid is concomitantly diminished giving rise to the so-called enhanced permeability and retention effect which allows NP to enter the tumor tissue from the blood stream via the fenestrations and stay there for extended periods of time [52]. Retinal diseases such as proliferative DR or wet AMD are likewise accompanied by neovascularization and increased blood vessels leakiness with a disrupted BRB (figure 4). We, therefore, deem future research to utilize these disease-related changes for therapeutic purposes highly promising. Like in tumor therapy, NPs would be a perfect drug transporter into the diseased tissue via the leaky blood vessels. Even though this type of therapy seems highly feasible, further extensive studies are essential. Thus, it must be clarified to which extent diseases-related changes in the eye vasculature and RPE (the outer BRB) allow for NP extravasation. The missing lymphatic drainage in the eye needs also to be scrutinized. Furthermore, the exact place of NP deposition in the posterior eye is an essential and decisive prerequisite for the delivery of drug molecules to their target site for the treatment of DR and other retinal diseases. Thereby, the main factors determining the deposition are the size and surface properties of the NP.

## Assessment of toxicity to pave the way to the clinic

NP have the potential to be a suitable and promising delivery system for drug molecules in ophthalmology. Nevertheless, the toxicological profile of particles is of crucial importance for a possible clinical application. Therefore, a comprehensive and intensive toxicological assessment is required as part of nanoparticle development. Currently, there are only a few reports on this issue in the literature. In earlier studies, toxicity has often been assessed in animals. However, the anatomical and physiological structures and properties sometimes differ significantly between humans and respective animal models. On the other hand, advanced techniques in the field of three-dimensional cell culture seem to offer new but still limited possibilities to mimic the human organ. Due to the limited amount of data, it is hardly possible to draw general conclusions regarding the toxicological profile of nanoparticle technology and to conclusively assess its toxicity [32].

While the data on NP toxicity in the literature is controversial, some potential toxicity mechanisms are outlined in figure 3H [32,53]. NP can have a toxic effect due to the dissolution of materials such as released ions [54]. Their binding to proteins and enzymes can significantly impair their functionality and have a negative impact on cell function [54,55]. Further damage is possible through the interaction of the metal ions with phospholipid membranes or genetic material of an organism [54,56] and oxidative stress [54,57]. This can impair important enzyme functions or lead to interaction with DNA whereby the type of ion plays a central role [54]. The generation of ROS and the subsequent oxidative stress is another toxicity mechanism, as it enables the damage of important enzymes or genetic material. The formation of ROS is possible through the NP itself or via the activation of signaling pathways that cause oxidative stress [54]. Lipid peroxidation makes a significant contribution to ROS formation [54,58]. ROS can attack various key enzymes such as mononuclear iron proteins and oxidize the bases and desoxyribose of DNA, thereby damaging the organism [54,59–61]. Furthermore essential and decisive factors for toxicity are the type of NP, the material it is made of, the size and shape, the surface charge and the coating of the NP [32,35]. In one study, the toxic effect was shown for polycaprolactone (PCL) and PLGA polymer NP in cell culture experiments. In the same study, it was shown that the cytotoxicity of NP can be reduced by PEGylation of NP in vitro. Therefore, a surface modification of the NP seems to result in reduced toxicity [35]. The use of an organic solvent-free manufacturing processes is definitely an advantage. Ultimately, toxicity plays a major role in future research of nanocarriers.

## Concluding remarks

NPs as drug carrier for ocular drug therapy have incomparable and promising characteristics. They allow for the transport of drugs that would by themselves not distribute in sufficient amounts into ocular tissue. While the large size of NPs is definitely a handicap for their ocular distribution, a careful surface design may help to overcome this limitation. Thus, addressing nanoparticles to cell surface structures that are characteristic for a target cell might help to deliver them to the cell of interest in the retina. In addition, pathophysiological changes of retinal blood vessels may allow NP to extravasate into retinal tissue where they could form a depot for drug release that allows for long term drug delivery.
